# Editorial: Novel design, synthesis, and environmental applications of covalent organic frameworks

**DOI:** 10.3389/fchem.2024.1434454

**Published:** 2024-06-06

**Authors:** Tanyue Chen, Anan Liu, Dongge Ma

**Affiliations:** ^1^ Department of Chemistry, School of Light Industry Science and Engineering, Beijing Technology and Business University, Beijing, China; ^2^ Basic Experimental Centre for Natural Science, University of Science and Technology Beijing, Beijing, China

**Keywords:** covalent organic framework, metal organic framework, environment application, materials characterization, novel methodologies, computational methods

The exploration of Metal Organic Frameworks (MOFs) and Covalent Organic Frameworks (COFs) have surpassed traditional boundaries of material science, offering innovative solutions to a range of global challenges from environmental pollution to the detection of risky substances. This editorial aims to encapsulate the recent strides made in the characterization and application of these versatile materials, while also highlighting methodological advancements that are propelling the field forward.

## Advancements in material characteristics and applications

Recent research of Guo et al. into the electrochemical behavior of chiral-doped Fe (Sudik et al., 2005; Scherb et al., 2008; Chen et al., 2017; Millange and Walton, 2018; Le et al., 2019; Navarathna et al., 2019; Ajpi et al., 2023) and Zn-based MOFs (Altaf et al., 2018) has unveiled significant insights into their potential as advanced materials for electronic applications. The findings indicate that the electronic and structural properties of these MOFs are predominantly influenced by the type of metal center rather than the chemical nature of the chiral dopants. This revelation was established through solid-state electrochemical measurements complemented by infrared spectroscopy, X-ray diffraction, and absorption techniques to further characterize their properties.

Chiral-doped MOFs, such as MIL53 S-CSA, have shown promising results in photocatalytic applications, particularly in water splitting for oxygen evolution reactions (OER). The enhanced photocurrent and efficiency observed in these materials can be attributed to their improved light absorption and catalytic properties. This makes them highly suitable for applications in sustainable energy solutions and advanced electronic devices.

The research also explores the impact of different organic ligands and synthetic conditions on the stability, porosity, and electronic properties of MOFs. By carefully selecting and manipulating these factors, researchers can fine-tune the MOFs’ characteristics to meet the demands of various specialized applications, including chiral recognition, separation and catalysis.

In summary, the development of chiral-doped MOFs represents a significant step forward in the field of materials science. By leveraging both experimental and theoretical approaches, researchers are unlocking new potentials for these materials in electronic and photocatalytic applications, paving the way for innovative solutions in technology and energy sustainability.

Ma et al.’s advancements highlight COFs’ potential as multifunctional photonic materials. Incorporating fluorophores into COFs has led to luminescent COFs with superior fluorescence compared to traditional organic solids. Their porous structures host various guest molecules, reducing internal friction, vibrations, and thermal losses that usually quench fluorescence.

The article highlights the application of fluorescent COFs in the detection and monitoring of explosive chemicals, showcasing their potential as chemical sensors (Das et al., 2015). The design principles and examples provided pave the way for future innovations in this field. Additionally, the article discusses the challenges of achieving fluorescence in two-dimensional (2D) (Dalapati et al., 2013) and three-dimensional (3D) COFs (Lin et al., 2016), such as overcoming aggregation-caused quenching (ACQ) in 2D COFs (Dalapati et al., 2013) and the scarcity of suitable fluorescent building blocks for 3D COFs (Lin et al., 2016).

Fluorescent COFs are promising for optoelectronics, energy storage, adsorption, separation, and catalysis due to their tunable structures. Incorporating aggregation-induced emission (AIE) concepts, as seen in TPE-Ph-COF (Dalapati et al., 2016) and 3D-TPE-COF (Smith et al., 2017), has boosted their fluorescence intensity and photoluminescence quantum yield (PLQY).

The article reviews fluorescence in COFs, focusing on π-π stacking, conjugated structures, stacking modes and fluorescent groups. These factors are critical for improving COFs’ fluorescence and applications, making them competitive with organic semiconductors. Ongoing research aims to unlock new functionalities for fluorescent COFs.

## Innovative research methods

In a groundbreaking study by Kriesche et al., researchers have combined ANI-2 Neural Network Potential (NNP) with molecular dynamics (MD) frameworks to explore the CO_2_ adsorption properties of COFs: HEXCOF1 (Alahakoon et al., 2016) and 3D-HNU-5 (Guan et al., 2019). Both COFs share the same linking unit, yet exhibit distinct structural configurations and CO_2_ adsorption capabilities (Alahakoon et al., 2016; Guan et al., 2019). HEXCOF1 features a two-dimensional layered structure, while 3D-HNU-5 boasts a three-dimensional tetrahedral geometry, forming an interpenetrated diamond-like topology (Alahakoon et al., 2016; Guan et al., 2019).

The study chose these structurally similar COFs to compare CO_2_ storage in 2D and 3D environments. Evaluating ANI-2 NNP’s performance before loading CO_2_ provided insights into how dimensionality affects CO_2_ adsorption in the COFs. The study shows that combining computational methods with experimental data enhances our understanding of COFs’ adsorption properties, aiding in the design of more efficient COF-based materials for environmental applications.

## Emerging applications in food safety

Guo et al.’s researches highlight MOFs’ potential in food contamination adsorption and detection. With large surface areas, unique pore structures, and versatile modifications, MOFs enhance food safety. Cost-effective zinc, copper, and zirconium-based MOFs effectively adsorb pollutants and serve as sensitive sensors.

MOFs’ selective adsorption improves sample purification and detection efficiency, leading to novel, rapid, portable, and cost-effective detection methods. They summarize these advancements, emphasizing MOFs’ ability to address traditional food safety challenges like high labor costs, expensive equipment, lengthy detection times, and complex sample preparation (Liu et al., 2019; Chen et al., 2022; Fu et al., 2022; Ghiasi et al., 2022; Majd et al., 2022; Zhang et al., 2022).

Despite these promising developments, MOFs’ integration into food safety protocols faces hurdles, including stability and selectivity Research Topic in complex matrices. Addressing these challenges through continued research and optimization will pave the way for MOFs to become indispensable in ensuring food safety, offering innovative solutions to one of the most pressing global concerns.

## Challenges and future directions

Despite these promising developments, the field faces several challenges. The scalability of synthetic methods, the long-term stability of MOFs and COFs under various environmental conditions, and the economic viability of these materials are areas that require ongoing research. Addressing these challenges will be crucial for transitioning MOFs and COFs from the laboratory to actual environmental applications.

## Conclusion

As the research community continues to explore the vast potential of MOFs and COFs, it is vital that we maintain a focus not only on advancing fundamental science but also on applying these materials to solve real-world problems. The convergence of innovative material properties with practical applications promises to drive the next wave of material science breakthroughs, potentially revolutionizing industries and improving global living standards. For researchers and students in the field, the journey is just beginning, and the opportunities to make a significant impact are boundless. As we advance, let us ensure that these materials are developed responsibly, with a clear vision towards sustainability and society benefit.
